# Substance use and help-seeking barriers: a qualitative study of East African migrants’ experiences of access to Norwegian healthcare services

**DOI:** 10.1186/s12913-023-09110-6

**Published:** 2023-02-02

**Authors:** Ruben Jervell Pettersen, Jonas Debesay

**Affiliations:** Faculty of Health, Department of Nursing, Oslo Metropolitan University, St. Olavs Plass, P.O. Box 4, NO-0130 Oslo, Norway

**Keywords:** Health-seeking behavior, Health disparities, Substance use, Migration health, Interpretative phenomenological analysis

## Abstract

**Background:**

Migration to Norway has increased rapidly in recent decades. Migrants have a lower prevalence of substance use, but may have an elevated risk of developing mental health issues and substance use problems due to various migration and post-migration factors. Few studies have sought to understand substance use problems among migrants in Norway. This study aimed to explore how people of East African background experience help-seeking for substance use problems in the Norwegian healthcare system.

**Methods:**

Using an explorative approach, in-depth individual interviews were conducted with six adult participants from Somalia, Eritrea and Sudan who had been in contact with the Norwegian healthcare system. The goal of the interviews was to facilitate in-depth and nuanced descriptions of the participants’ lived experience of help-seeking for substance use problems. The data were analysed using interpretive phenomenological analysis.

**Results:**

The analysis resulted in five themes in which participants described their help-seeking experiences for substance use problems as lack of knowledge and access to information, scepticism towards a ‘white system’, fear of exclusion from family and ethnic community, racism as a barrier to help-seeking, and positive experiences and ideas for future treatment practices.

**Conclusion:**

This study provides an improved understanding of how migrants with substance use problems experience help-seeking in healthcare. The variety of barriers illustrates inequality in substance use care for East African migrants in Norway.

## Background

The harmful use of substances is a major global health concern and is recognised as one of the top 10 disease burdens worldwide [1]. Alcohol, the most commonly used substance, contributed to 3 million deaths globally in 2016, ranking the mortality associated with alcohol consumption higher than diseases such as tuberculosis, diabetes and HIV/AIDS [2]. According to the United Nations Office on Drugs and Crime, 35.6 million people worldwide suffer from drug use disorders, while only one in eight receives treatment [3]. It is estimated that 20% of people with a severe mental health disorder will develop a substance use disorder during their lifetime. However, only 7,4 percent receive treatment for both disorders, and 55% receive no treatment [4]. Despite increased attention in recent years, there is still a dearth of research on substance use among migrant populations, especially forced migrants [5]. Research refers to many types of migrants, such as labour migrants, student migrants, internally displaced people, refugees and asylum seekers. In this article, the focus is on migrants who, in different ways, have been forced to relocate because they are fleeing from conflict or persecution, seeking protection or have status as refugees [6]. When speaking of substance use problems in this article, we refer to the WHO definition of “harmful use” [7]:

A pattern of psychoactive substance use that is causing damage to health. The damage may be physical (e.g., hepatitis following injection of drugs) or mental (e.g., depressive episodes secondary to heavy alcohol intake). Harmful use commonly, but not invariably, has adverse social consequences; social consequences in themselves, however, are not sufficient to justify a diagnosis of harmful use.

A systematic review of substance use among forced migrants globally reported significant variations in prevalence. The included studies using validated measures reported prevalence rates of alcohol dependence ranging from < 1%–42% and drug dependence ranging from 1%–20% [5]. The authors stated that such heterogeneity in substance use patterns makes it hard to draw definitive conclusions and is likely contingent on many factors, including local contextual aspects, such as substance availability and social norms. More importantly, the results may sometimes underestimate the exact prevalence, because stigma has been documented to affect the reporting of substance use in culturally diverse communities [5]. A recent systematic review of qualitative research on substance use among refugees concluded that refugees have an elevated risk of substance use and developing substance use disorders. Substance use as a coping mechanism or self-medication among refugees has been documented in studies from several regions around the world [8].

Migration to Norway has nearly quadrupled in the last two decades. At the beginning of 2022, 819,356 migrants lived in the country, amounting to 15.2% of the entire population [9]. Half of all migrants have lived in Norway for less than 10 years [10]. Migration and post-migration stressors, such as difficulties in handling cultural changes of dislocation or experiences of discrimination and racism, may contribute to adverse health outcomes over time, especially in high-income countries [5]. In Norway, there is very little available knowledge on substance use in migrant populations, but there are indications of lower consumption compared to the majority population. For example, the proportion of migrants who have consumed alcohol in the last year is estimated to be significantly lower, especially among migrant women [10]. According to a national survey on living conditions among migrants in Norway, 17% of migrant men and 11% of migrant women reported having consumed alcohol in the last week, compared to 44% and 33% for men and women, respectively, in the general population [11]. A cross-sectional study among adolescents in Oslo found a lower risk of binge drinking and cannabis use among those with ethnic minority backgrounds compared to ethnic Norwegians. This risk was particularly reduced among adolescents from the Middle East, Asia and Africa [12]. Migrants from Africa make up a small, but fast-growing part of the Norwegian population. Half of all migrants from Africa have arrived in the last decade, and the majority hail from East African countries [9]. To our knowledge, this is the first study exploring substance use problems and help-seeking among East Africans in Norway.

### Barriers to healthcare access in migrant populations

Barriers to accessing healthcare services in migrant populations are well documented [8, 13, 14]. The barriers exist and intersect on multiple levels [15]. Some may be related to the individual, such as distrust, linguistic challenges and lack of health literacy, while some are more systemic, such as lack of relevant expertise and resources among healthcare personnel, referral bias and poor implementation of diversity policies. For instance, being denied treatment because of low language proficiency (systemic) can create distrust towards health services in the individual [15]. With increased migration levels worldwide, it is essential to understand the barriers that may affect help-seeking in resettlement countries [13].

A systematic review of quantitative and qualitative studies found that stigma was ranked the fourth highest barrier to help-seeking for mental health issues. Ethnic minorities, together with men and professionals in the military and healthcare, were identified as population groups that are disproportionately affected by stigmatisation [16]. Along the same lines of the exclusionary practice of stigmatisation, there is growing evidence of the negative role that discrimination and racism play in substance use and access to treatment [17]. Research has shown that ethnic minority populations may experience disparities in help-seeking for substance use problems, are more likely to receive delayed treatment and have lower retention rates than majority populations [18].

Multiple barriers to accessing health services for people with migrant backgrounds have been documented in Norway [10]. A lack of health information in other languages and perceived discrimination and racism from healthcare personnel have been found to make healthcare services less attractive among sub-Saharan African migrants [19]. A study exploring Norwegian general practitioners’ consultations with refugees showed that language barriers, different understandings of health and illness, mismatched expectations and feeling unprepared to work with the patient group negatively affected patient encounters [20]. Research on help-seeking for substance use problems among migrants is particularly scarce in Norway. The limited research available highlights a lack of culturally competent care in Norwegian substance use treatment [21, 22]. While migration contributes positively to the host society [23], and demographic changes and increased sociocultural diversity have many advantages, it also presents challenges to providing high-quality services by the welfare system [24]. Norway aims to offer equal access to healthcare services for all its citizens, but it is still far from achieving the goal of health equality in substance use care [25]. In line with the ambition of equal access to care, it is crucial to gain more knowledge about substance use and help-seeking in different migrant groups to develop targeted public health efforts and reduce inequality in health.

### Aim

This study aimed to explore how people of East African background experience help-seeking for substance use problems in the Norwegian healthcare system.

## Methods

The study design is qualitative, explorative and predominantly inductive in its approach to elicit the participants’ experiences of help-seeking for problem substance use. However, the researchers’ pre-understanding of theories such as discrimination, stigma, racism and health literacy also influenced planning, data gathering and interpretation of results. The methodology can therefore be conceptualised as abductive in nature, employing a combination of inductive and deductive reasoning [26]. The term ‘help-seeking’ used in this article includes all stages of the process of initiation and engagement with care [16]. We chose interpretative phenomenological analysis (IPA) as our methodological framework. IPA follows three guiding principles [27]:

1. The study must examine the lived *phenomenological* experience of the participant.
2. It requires a rigorous *interpretative* effort through a dual-layered process in which ‘the participant is trying to make sense of what is happening to them while the researcher is trying to make sense of the participant’s sense-making’ [27, p.8].
3. The *idiographic* focus demands attention to the individual and the experiences of each participant.

### Recruitment

The recruitment of study participants was purposive, but considering the stigma associated with the topic (substance use) in combination with the group of interest (East Africans), recruitment proved challenging. The idiographic approach and modest requirements for sample size make IPA suitable for stigmatised or hard-to-reach populations [21]. Representatives from two interest organisations working with mental health and substance use among migrants assisted in recruiting participants. Both representatives were of East African background and knew their communities well. One of the representatives also had a prior history of problematic substance use. The interest group representatives provided a critical bridge of trust between the project and the participants. The representatives asked potential participants on behalf of the project and supplied contact information to the first author. The first author then contacted the participant to confirm interest, answer questions and schedule an interview.

We included people above the age of 18, born outside of Norway, with a national background from Somalia, Eritrea, Ethiopia or Sudan, with a self-reported history of problem substance use and experience with help-seeking in the Norwegian healthcare system. IPA’s focus on a specific experience requires a reasonably homogeneous sample [28]. In other words, participants need to be more similar than dissimilar to facilitate interpretation and capture the essence of the phenomenon in question. The specific country backgrounds were selected because they make up the majority of migrants from African countries in Norway with 65,6% [9]. Furthermore, dark skin colour makes the participants´ migrant status highly visible in a majority white society, as opposed to other migrant groups with lighter skin. Participants share a comparative similarity concerning the research question when it comes to forced migration background, short residence time compared to more established migrant groups in Norway and geographical and cultural proximity of native countries (see Table 1).

**Table 1:** Participant overview

Participants	Gender	Age	Total years of harmful substance use
Sophia	Female	40–50	10
Claire	Female	20–30	6
Patrick	Male	30–40	7
John	Male	30–40	20
Ali	Male	30–40	5
Yusuf	Male	40–50	10

### Interviews

We conducted six individual in-depth interviews between August and November 2020. An open-ended interview guide was developed and loosely followed to allow the participants to take the lead on subjects that were particularly important and meaningful to them. Participants in IPA studies are considered experts in their own experience. This opens up unexpected avenues of exploration that the researcher might not otherwise have anticipated [28]. Participants were asked to share their experiences of help-seeking from healthcare services for substance use problems, thoughts on why they used substances, whether the use of illicit substances affected them in any way, if they had experienced discrimination while seeking help and what was essential for them when asking for help. The interviews lasted between 60 and 120 min and were conducted by the first author. All interviews were audio-recorded, transcribed verbatim and participants’ names were replaced with pseudonyms.

To reduce power asymmetries and avoid socially desirable answers, the interviewer tried to facilitate a safe environment for the participants. The goal for each interview was to balance the need to produce rich data and keep the participant at ease while discussing delicate topics. The interviewer was conscious of communicating respectfully, showing empathy, and avoiding judgment. Participants were reminded before and during the interview that they decided which questions they wanted to answer and that there were no “wrong answers.” The participants were asked to suggest a time and place at their convenience or meet in a private office on university premises. Four interviews were conducted in the university office, one in a secluded room at a public library and one in the backroom of a café. Interviews were conducted in Norwegian. All participants except for one were fluent speakers of Norwegian at the time of the interviews. One participant had occasionally some difficulty expressing himself in Norwegian and therefore could not go into the same level of detail on all themes. This participant also chose to include a bi-lingual friend at the start of the interview, who assisted in translating the information required to give informed consent. After interviews, all participants were invited to share their overall experience of the interview and emotional state. The interviewer was prepared to escort participants to emergency psychiatric services, but this was not needed.

### Analysis

In adherence to IPA’s idiographic focus [27], a comprehensive first reading was conducted for each transcript before a cross-case analysis among the participants was performed (see Table 2). The analysis started with a careful reading and rereading of the transcript by both authors to gain familiarity with and immersion in the data. This was followed by exploratory note-taking, in which anything of interest was jotted down, whether descriptive, linguistic or conceptual [28]. Then, a comprehensive list of experiential statements was formulated for the entire transcript before they were clustered into named groups. Finally, a table of personal experiential themes was created. Upon completion of the personal experiential themes of all cases, a final synthesis of five group experiential themes was identified through points of convergence and divergence. The five experiential themes form the basis of the article’s results section. A description of the analysis process related to one of the study themes is illustrated in Table 3.

**Table 2:** Steps of IPA analysis (based on Smith and Nizza [2])

Step 1: Reading and taking exploratory notes	Slowly reading and rereading transcripts and going back to the audio recording when necessary to ‘evoke the participant’s voice’. Staying open, noting everything of interest
Step 2: Formulating experiential statements	Capture in a concise form the experiential meaning(s) of each portion of the transcript. A condensing effort allows both the participant’s and the analyst’s interpretations to unfold
Step 3: Finding connections and clustering experiential statements	Rearranging the complete list of experiential statements into clusters. Aiming to review and refine the analysis to show key features from ‘a bird’s eye view’ of the data
Step 4: Compiling a table of personal experiential themes	Once clustering feels satisfactory, each cluster is named as an overarching personal experiential theme. The experiential theme should relate to information on all the experiential statements in the cluster
Step 5: Moving to the next case	Repeating steps 1–4 for the next case. Respecting the idiographic focus by treating each case on its terms through ‘bracketing’ or trying as best as possible to avoid influence and association spilling over from the other cases
Step 6: A cross-case analysis	After each case has been individually analysed, it is time to establish connections and idiosyncrasies between cases, focusing on how each case can illuminate the other. The results are compiled into an overview of group experiential themes, which serves as the basis for writing up the findings

**Table 3:** Example of the analysis process

Quotation	Experiential statements	Personal experiential theme	Group experiential theme
*I have spoken about it with other Somalis, friends, and people the same age, and absolutely, there is a substance use problem in our community among older men […] there have been times where mom spoke about women who use khat and these women were described as people who have, like, left their faith, culture, disregarded their families. They were much harsher against them than their husbands, brothers and fathers, so yeah, I definitely experience shame because I get high. If I had told this to my mom, then I would be left with a feeling of shame, guilt and extreme uneasiness, like… I must prove that I am Somali, prove that I am Muslim and that I haven’t left all that behind. (Claire)*	Increasingly aware of substance use also being a problem in her own community Substance use among Somali women is more shameful and judged more harshly than among men A need to maintain an image as a good Somali and Muslim	Prejudice against substance use and mental health in community	Fear of exclusion from family and ethnic community

The sample for this study included six people, comprising four men and two women, with an average age of 37.3 years. The highest attained educational level was one undergraduate degree, four high school diplomas and one primary school. Country of origin were Somalia (4), Eritrea (1) and Sudan (1). The reported substances used were alcohol (*n* = 4), cocaine (*n* = 4), cannabis (*n* = 3), heroin (*n* = 3), amphetamine (*n* = 3) and benzodiazepines (*n* = 2). All participants reported previous or current use of at least two substances. Half of the participants reported injecting substances at some point.

Four participants had undergone treatment and were not using substances at the time of the interviews, ranging from 10 + years to 1 year of sobriety. One participant was about to enter a treatment programme for the second time and one had no treatment experience. Everyone shared some thoughts on the underlying reasons or catalysts that launched them into problem substance use. One told the story of how he broke down when his ex-wife took his children away from him. Two participants also told stories of loss, sharing the experience of grief from a dying parent or child. Another participant suffered from depression in her teens, prompting her parents to bring her back to Somalia to culturally rehabilitate her, with added trauma and anxiety as a result when she returned to Norway. The same participant also drew parallels between her father’s problematic khat consumption and her own substance use issues. One participant also underscored the role of trauma as a red line marking her life from having to flee alone from war as a small child to the insecurity, neglect and rejection she felt while she was moved around by child protective services for most of her upbringing. One participant first turned to weed, then alcohol, to cope with feelings of isolation, missing his family and lack of meaningful activities in the new country. Everyone described the self-medicating role that substances played in various ways as a response to difficult emotions and trauma.

### Ethics

The study was approved by the Norwegian Centre for Research Data (Project No. 704756). The research project was designed and executed following the Declaration of Helsinki. Participants were provided written information and an oral explanation of the project before the interviews, including collection, storage and handling of personal data. Ample time was given for questions before and after interviews. Participants gave informed consent to take part in the study and for the use of pseudonyms to replace their names in publications. Each participant received information on the possibility of withdrawing from the study at any time without stating any reason. Contextual and demographic descriptions are presented in terms as general as possible. The audio files and interview data were stored at the University of Oslo’s encrypted platform service for sensitive data (TSD). Participants were given the possibility to read through and comment on the submitted manuscript prior to publication.

## Results

The analysis resulted in five experiential themes: lack of knowledge and access to information, scepticism towards a ‘white system’, fear of exclusion from family and ethnic communities, racism as a barrier to help-seeking and positive experiences and ideas for future treatment practices.

### Lack of knowledge and access to information

A significant finding in the study was a lack of knowledge about substance use, mental illness in general and opportunities for treatment. Most of the participants expressed how difficult it was to find helpful information in their situation. Not knowing where to start left several of the participants in a passive and destructive state of uncertainty. Yusuf and John said that there was a long period before treatment, where although they might have been conscious of the severity of their issues, they were unaware of the possibility of receiving help:

*I knew all along that when I looked at myself from the outside, I didn’t like what I saw or the road it was leading to, because I could clearly see where this was going, right? [Sighs heavily] I didn’t know what else to do; I was simply drifting around town [...]. Yeah, it was hard [...], like, you decide to get help when you know that the help exists, right? But it took a very long time before I even knew that help existed out there, right? (Yusuf)*

*There was a lot I didn’t know, like, for example, my Norwegian buddy knew all along, but why didn’t I know? Yeah, so it could be that a lot depends on the family; maybe his family has more education, knows more, and because they are Norwegian, they know their rights and how to seek help. I didn’t have that at home at all; I had to figure it out by myself. And in school, you don’t get these things either [information about substance use and treatment]. (John)*

John perceived that much crucial information had never reached his family and community when he was growing up; he ascribed this to both linguistic and cultural barriers. He described how the children of first-generation refugee families must often serve as translators to help their parents navigate the new society. Several times, John contrasted his experience with that of his Norwegian friends who had more robust social networks and parents who were better informed to deal with issues of poor mental health and substance use. John believed that ‘knowledge is power’ and that the lack of knowledge rendered him and his family powerless in the face of his addiction. Patrick also noted that his knowledge about the Norwegian healthcare system as a younger man was ‘*zero*’ and so limited that he did not understand what the term *mental health* signified. Despite high proficiency in Norwegian, grasping mental health terminology was mentioned as a challenge for most participants.

During the interview with Ali, who had lived in Norway for a shorter period than the others, we saw how this unawareness was evident. Ali asked how he should proceed to receive help for his drinking problem. When asked what type of help he thought he needed, Ali was uncertain and asked the interviewer what he thought would be best for him. He was unaware of established self-help communities such as Alcoholics Anonymous. Nor did he not know that he could be referred to services from his GP or that he would be safe to contact occupational health services at work. Hence, Ali posed questions that he thought could help him explore options and help him move forward.

### Scepticism towards a ‘white system’

Several participants expressed feelings of scepticism and distrust towards what was sometimes labelled the *white system.* In other words, the participants positioned themselves as outsiders from a majority white healthcare system that was not created for people with a different ethnic, cultural, religious or social background:

*There is so much difference [between her and the health system], all at once, so it becomes sort of like, ‘Okay, this is not even worth bothering to try to sort out’. (Sophia)*

*I’m left with a feeling of inner turmoil [after treatment] when it comes to questions of family, religion and sexuality. What will I do moving forward if having substance use issues is a lifelong struggle? A problem I will have to keep in check, in addition to all these other things that I’m feeling. I don’t feel like my inner anxiety has been reassured. (Claire)*

In most encounters with healthcare workers, the inexperience with minority ethnic patients was tangible to the participants; this led to frequent misunderstandings, frustration and unmet needs. Ali had, on one occasion, unsuccessfully asked for help from the GP he was assigned to in the first city he had settled in after leaving the asylum centre. The experience left him feeling sceptical and unsure about what he could expect:

*What can the GP do? The GP only does nothing [...]. I know from before [...] I met with the GP he told me, ‘You drink much, you have problems, you have stress’. I said, ‘Yes, I have problems with family and thinking so much’. He [the GP] told me, ‘Don’t think so much, just go to the gym or exercise’. Because I went to him, I don’t talk with the GP [anymore] (Ali)*

Feeling immediately dismissed and left to his own devices, Ali gave up on pursuing help from the healthcare system. Since the GP’s appointment a few years earlier, it appeared as if he had not discussed his issues with anyone except a few members of his family and some friends. He seemed to struggle with the thought that his alcohol problems were his responsibility and his fault alone. Ali was not alone among the participants in feeling sceptical:

*John: I was pretty sceptical when I came into this [treatment programme]; they use something called a ‘community method’ [...]. So yeah, there were no Somalis, at least not at that time, or people with dark skin among those who worked there, so I was sceptical about how I was going to be treated. [...]And then I think, ‘How am I going to be treated? Will I be treated worse than the others’? (John)*

John was not the only patient in this treatment community who worried about how they might be received, but he explained that his wariness was due to his skin colour. John’s previous negative experience with the police gave him reasons for his suspicions; he felt he had been disproportionately targeted by law enforcement due to his dark skin. He had been beaten up by law officers at one point to such an extent that they broke his shoulder. These experiences spoiled his impression of public services, including healthcare. Some of the male participants described difficulties perceiving the separation between police and healthcare. They also knew about people in their communities who were deported because of drug involvement. When asked if fear of punishment somehow affected his willingness to seek help, Patrick replied:

*Both yes and no, I would say. I was afraid to be prosecuted by the authorities because I had only heard bad things about the police, and quite frankly, I had a lot of prejudice. I had put everyone in the same category: help services, police, everything. (Patrick)*

Patrick and Yusuf explained how they carried a deep-seated distrust of authorities from their native country, where different institutions were much more porous and weakened by corruption. They believed that the police in particular were not to be trusted, and if they were involved in illegal substance use, there was no guarantee that healthcare providers would honour their confidentiality.

### Fear of exclusion from family and ethnic community

Although the study participants recounted some of the more commonplace feelings of shame and humiliation surrounding substance use in society, they revealed that traditions, cultural beliefs and stigma associated with substance use in their communities played a crucial role in preventing them from seeking help:

*My reputation in my community, my friends, the face of my family, and the clan I come from, right? All that, it’s going to go like, ‘Yusuf, now you have fucked up for all of us’ [nervous laughter]. So, there was a lot at stake. I was afraid for my family, for everyone, honour and pride—all that is at risk, you know? (Yusuf)*

Substance use was experienced as taboo for all the participants, and openly turning to the healthcare system for assistance would be to admit weakness and defeat. It felt difficult to discuss their challenges in familial settings. Even if some participants suspected that family members were aware, the problem remained undiscussed. No one close to them ever approached the subject, at least not in a direct fashion. Steering too far from their community’s expectations could pose serious consequences, such as being unworthy of marriage or facing total exclusion:

*What I was most afraid of was my relationship with my family. How were they going to take it, me being a criminal and drug user, right? [...]. To put it like this: talking about drug use within the family, traditions and that, if a person is an alcoholic or a drug user, then it’s a person who is lost or a lowlife in a way [...]. They wouldn’t say it to me, but nobody in your family would go out and say, ‘Oh, yeah I have a brother who’s a drug user’ [...]. They want to keep the secret as best as they can. You could say that the Somali culture is not as open as the Norwegian culture. (John)*

Women who used substances could expect harsher judgment in their communities. For instance, the consumption of the stimulating substance *khat* among men in Somali culture was widely accepted. On the other hand, women who chewed khat were described as having left their Islamic faith and culture and neglecting their families. The more common substances consumed in Western countries would be even worse. At the heart of Claire’s struggles lay the constant pressures of living a double life. From being what she labelled a ‘*model Somali girl*’ in her upbringing, she was now balancing on a knife edge, getting high to alleviate her stress. At the same time, that same behaviour could potentially cause her biggest fears to materialise:

*My biggest fear is rejection [...] not dying, not that someone I care about dies, just rejection from someone I love [...]. Yeah, I’m terrified of rejection, so what substances gave me was that I didn’t have to think so much about it, and also the lifestyle that comes with it and partying, it sort of gave me a break so that I didn’t have to think about these things and for a moment, not be so afraid of rejection. (Claire)*

Several participants imagined that revealing their substance use problems could produce a worst-case scenario of rejection and a lack of belonging. They expressed a fear of finding themselves alone between cultures. Sophia knew what this could look like:

*The most marginalised people in Norway, that’s people like me. Not a part of my native culture, not a part of Norwegian culture, heavily traumatised. (Sophia)*

When she migrated to Norway as a small girl, Sophia was reunited with her father, who was her only relative and who eventually proved incapable of caring for his daughter. Years of different foster parents and group homes ensued until she was left to live on her own while still a teenager in high school. For most of her life, Sophia was therefore left in the worst-case scenario that the other participants feared: a vulnerable void of rejection and exclusion.

### Racism as a barrier to help-seeking

Discrimination and racism in treatment were major challenges to the participants. They shared repeated, lifelong experiences, ranging from bullying in school to being called racial slurs and spat at while walking down the street as adults. The damages of racism, in all its forms, were an essential part of their emotional baggage, carried around wherever they went, including treatment. Although they were placed within the confines of institutional care and protection, they were not protected from racism. Participants described how they were reminded of their differences everywhere they turned and sometimes suffered greatly because of it:

*And then they just start to talk about Africans and yeah slaves and negro and, like, it affects you. It feels uncomfortable, and even if you can’t say that it’s racist, what’s being said or how others are using their words, but for me, it feels really uncomfortable to be sitting there. [...] So yeah, there was a healthcare worker who enjoyed using that word [negro] all the time, and meant that was the correct way to say it, so [...]Yeah, and then we were twenty people, all were white, and the only one with dark skin was me sitting there, and he said that word again and again, and then I felt that it became too uncomfortable for me to sit there. I spoke up and told him I didn’t want to listen to him if he was going to talk like that. He [healthcare worker] responded, ‘But that’s just how we talk’, I said, ‘Okay’, and I left. (John)*

There were many stories of racism in treatment. On a different occasion, John and another patient of African origin were encouraged by fellow white patients to make monkey sounds in an offensive attempt at humour at their expense. John admitted to feeling so uneasy from discriminatory situations that he experienced physical nausea and almost left treatment. Moreover, the heavy emphasis on group therapy in the care system proved to be challenging. All participants with treatment experience shared negative feelings and insecurity associated with being the only black person in the room. Yusuf expressed how he was hypervigilant, always ready to protect himself against the white people he felt were ‘*out to get him.*’ This was not helped by the fact that his patient group had an outspoken, far-right extremist in its midst. At one point, Yusuf and the far-right extremist got into a dispute, and Yusuf was the one who nearly got kicked out of treatment because of it, putting his recovery at risk.

Not all experiences of racism had the direct interpersonal nature of John’s and Yusuf’s examples, but that did not mean they were any less damaging. Sophia reasoned that her entire upbringing as a lonely and neglected black child in the system could be considered a form of discrimination. Furthermore, she felt that being open about previous substance use and having a minority background hindered her ability to see a psychologist. After numerous refusals, it reached the point where she had to ask a social worker friend to give her a recommendation to be accepted for counselling:

*It’s really hard to find a psychologist with a reimbursement scheme [...]. Even after I quit substances altogether, I thought it would be very easy to get help. I got a list [of psychiatrists] from my GP at the time, and I was so naïve! I didn’t know that you should never even think about writing that you have a history of substance use [frustrated laugh] and preferably not that you are a minority. I mean, I didn’t know I was the type of patient you were least likely to want [in treatment]. (Sophia)*

Participants also spoke of events where the intentions of healthcare personnel were probably good, but nonetheless upsetting. Before a group session, Claire was asked by a nurse if she could explain how she experienced racism in society. Feeling unable to say no, she agreed to act as a ‘*token black girl*’ due to the asymmetrical therapeutic power relation and an intrinsic feeling of responsibility to enlighten her fellow [white] patients. The problem seemed not to be the request itself, which Claire admitted she could have appreciated in a different setting, but having to explain racism at a vulnerable point in life, where the last thing she needed was a spotlight, accentuating her difference even more.

### Positive experiences and ideas for future treatment practices

Despite many difficulties and delays in service uptake, many of the participants expressed gratitude for the help they received in the end. This was especially true for more universal challenges, such as dealing with the deaths of family members or gaining a fundamental understanding of the mechanisms and pitfalls of addiction. Several told stories of good fortune, where they serendipitously crossed paths with a healthcare professional who was patient, flexible, curious enough to gain their trust, knew how to ask the right questions and empowered them to act:

*Patrick: It was a really good meeting, and I feel that he sort of met me in a humane way. [...] Because he kind of asked me what I wanted to do. He put the ball over in my corner and asked me, ‘Well, what do you want to do with your life?’ […] And what that did to me was that it got my mind going […] That he put the ball in my corner and said, ‘What do you want to change? What kind of changes do you want to make to improve your life from the situation you are in right now?’ And it was precisely what I came there to achieve. And I didn’t know that the answer was inside me all along. (Patrick)*

Meeting a person ‘*who saw me for who I am*’ was deemed a crucial turning point on their path towards recovery. The participants did not mention whether these professionals possessed specialised competency in working with East African patients. The healthcare workers’ ability to come through to the participants seemed to lie elsewhere in that they were respectful, believed in the participants’ capabilities, and simply acknowledged the person in front of them. Unfortunately, the positive encounters were described as exceptions and not as the rule in the participants’ experience. Feeling misunderstood or disregarded was more common:

*It’s a form of not being seen, I suppose. I believe that many think, ‘Oh well, if you’re a refugee or were raised in child protective services, yeah, sure, you’ve probably experienced a lot of bad stuff, but that sort of comes with the territory for people like them’ [frustrated laugh]. The feeling I’ve often been left with is that people think, ‘Pretty and dainty [white] middle-class people shouldn’t experience anything bad’. So if they have, it’s like, ‘Oh my God, how horrible for that person’, but for the likes of me, it’s expected. (Sophia)*

When asked about what they would like to see improved in how healthcare receives patients with a minority ethnic background, some participants were a bit reticent initially, admitting somewhat restrained expectations of change. However, they all presented several suggestions when offered to think of a dream scenario:

*Wishful thinking isn’t always fun, but let’s say it’s hypothetical and my first time entering treatment. I go in, and there’s a certain sensitivity towards the fact that I don’t want my family involved in treatment, which is respected! Without digging further into it like, ‘Oh, but it’s good for you [to include family] blah blah’, without acknowledging that it’s hard, it’s really hard. And I would like to have a person there who has the experience, specifically with minority backgrounds, specifically Muslim/Somali, and I would also have said yes to a family group therapy service where there’s an interpreter. I do know that I’m entitled to it, but not just an interpreter, but rather a skilled professional who can understand my situation and mediate. (Claire)*

More awareness, inclusion and cultural competence in healthcare were chorused. Some participants also shared Claire’s wish for more people with ethnic minority backgrounds to act as cultural brokers in treatment. Furthermore, the importance of increased trauma competence among healthcare workers in substance use care was emphasised by some participants. Participants wished for more awareness and knowledge of traumatic experiences such as fleeing war, racism in the new country or failed attempts to return to the native country for cultural rehabilitation (otherwise known as *dhaqan celis* in the Somali language/tradition).

## Discussion

This study aimed to explore how people of East African background experience help-seeking for substance use problems in the Norwegian healthcare system. The main finding was that the participants encountered multiple barriers to seeking help. Barriers were experienced before and during substance use treatment, resulting in delayed treatment uptake and perceived inequality in service outcomes. The interviews revealed lower levels of health literacy, community stigma towards mental health issues and substance use, perceived racism, lack of trust in services, and absence of cultural competency among healthcare professionals.

Lack of information and low health literacy about mental healthcare were experienced as significant barriers to substance use care by the study participants. With limited comprehensible and readily available information about mental health, substance use and help services, it is unsurprising that help-seeking in some East African communities is affected negatively. Here, it is worth mentioning that the Norwegian Integration Act, which instructs the role, content and function of the introductory programme for newly arrived migrants, does not specify knowledge of health or healthcare services in its requirements for the programme’s minimum curriculum content [29]. The emphasis on health subjects, including mental health service utilisation, is therefore mostly left to the discretion of each municipality. Although all participants in our study spoke Norwegian, some admitted that the vocabulary required to understand mental health jargon was challenging. Most of them had lived in minority communities where high-level proficiency in the Norwegian language was less common, contributing to reduced circulation and discussion of relevant health information. In a study of access to healthcare among sub-Saharan African migrants in Norway, language proficiency was discussed, emphasising a need for better information about the healthcare system, illness and preventive measures in English and languages other than Norwegian [19]. A recent systematic review on the health-seeking behaviours of migrants, asylum seekers and refugees in Europe is consistent with our findings, revealing that many migrants across Europe are unaware of services and how to access them [30]. Similar findings have also been reported in North America and Australia [13, 14].

Stigmatising attitudes surrounding mental health issues and substance use were identified as major challenges for the participants. Stigma has been widely discussed in relation to mental health help-seeking, but there is still much left to be understood [16]. For example, quantitative findings on the association between stigma and mental health help-seeking among military personnel have been shown to be inconclusive [31]. On the other hand, the qualitative literature suggests that there is strong evidence of the negative role of stigma in help-seeking [32]. The participants in our study disclosed a fear of being negatively perceived by their communities if they openly asked the system for help. Even if they want help, members of some migrant communities can risk bringing shame and humiliation on their entire families or clans [21, 33]. A Canadian study exploring access to and utilisation of mental health services for migrant and refugee patients from the perspective of service providers found the persistence of stigma to be one of the main barriers. Participants described how migrant and refugee patients often felt the need to conceal mental illness from family and support networks, which in turn increased isolation and amplified mental health issues [34]. A recent systematic review of qualitative studies exploring substance use among refugees presented stigma as a psychological and social barrier, while stressing that substance use is also gendered. Substance use among female refugees was found to be more stigmatised than among men, highlighting how stigma can contribute to gender-based inequality in substance use treatment [8]. The increased stigma and fear of detection among migrant women with substance use problems have previously been hypothesised in Norway [21]. The results of our study indicate that the gendered aspect of stigma may play an important role in help-seeking for substance use problems in certain migrant groups.

Our research data showcase the perceived detrimental effects of racism on the participants’ mental health and help-seeking experiences. The experiences were both interpersonal and systemic, negatively affecting access to services and the perceived quality of treatment. Acknowledging racism and preventing maltreatment and inequality associated with skin colour are often dismissed in public discourse in majority white societies, and Norway is no exception. Migrants are often represented through a hostile and problematic lens in public discourse and, to a lesser degree, as a resource for society [35]. A survey conducted in 2020 revealed that one in three Norwegians does not believe racism is a problem [36]. However, evidence suggests that discrimination and racist exclusionary practices are widespread across multiple arenas of Norwegian society. Ethnic minority youth, especially males, experience disproportional ‘stop and searches’ from the police [37] and have been overrepresented in cannabis crime statistics in Oslo, even though cannabis use is higher among majority ethnic Norwegians [38]. In the employment and housing market, citizens with foreign-sounding names are significantly less likely to access job interviews and rental housing [39]. Furthermore, a recent UNICEF report investigating racism among Norwegian teenagers revealed that 37% had been subjected to racism due to their skin colour, with school being the worst arena for more than half of the respondents [40].

In Norwegian healthcare, patients with a minority ethnic background consume fewer healthcare services overall. This includes mental health services, despite reporting higher levels of mental health problems than the general population [41, 42]. When the pandemic was in its infancy, some voiced the idea that COVID-19 did not discriminate. These voices quieted down as people with minority ethnic backgrounds in Norway and across Scandinavia became heavily overrepresented in terms of both positive cases and hospitalisations [43, 44]. Disproportionately adverse outcomes from the pandemic have been documented in racialised communities worldwide [45, 46]. The pandemic serves as a bleak reminder of the pervasive and systemic nature of racism and how it acts as a fundamental cause of inequality in healthcare [47]. Following African American George Floyd’s brutal death and the stark racial health inequalities in the aftermath of the pandemic, more researchers are now arguing that racism should be given more attention in public health [46, 48, 49]. Interpersonal and systemic racism remain severe impediments to service access, satisfaction and completion within the field of substance use treatment as well [17]. We stand behind the argument of racism as an important public health issue among certain migrant groups and find that our study’s results contribute to the growing body of evidence in its favour.

A perceived lack of cultural competency and personalised treatment in Norwegian substance use treatment is in line with previous research on migrants with co-occurring substance use and mental mealth disorders [21, 22]. Furthermore, the challenges of transcultural care are mirrored in studies from the perspective of healthcare providers. One study of healthcare workers’ encounters with ethnic minority patients revealed that professionals might feel anxious about the unfamiliarity of treating patients from a different cultural background, experience prejudice towards patients and fear being incorrectly labelled as racist [50]. Another study reported transcultural challenges among healthcare personnel comparable to those mentioned above, while also emphasising the importance of curiosity and flexibility to improve patient–professional relationships [22]. Curiosity and flexibility among healthcare workers fit well with our study participants’ descriptions of successful patient–professional encounters, contributing to a feeling of being treated ‘as a person’ [21]. Participants expressed a need for more cultural competence, and much research supports their suggestion to improve access and service provision for ethnic minority patients [21, 22, 51, 52]. While cultural competency in healthcare providers is likely to smoothen interpersonal therapeutic relationships, its efficacy in reducing health inequalities on its own has been questioned [53]. In fact, evidence on the development of substance use problems and access to mental health services suggests that culture is not the main issue in overcoming health inequalities [54]. Moreover, cultural competency has been criticised for putting too much responsibility on the individual in healthcare services while ignoring more structural considerations and policy-level barriers [15, 50]. Therefore, to reduce treatment access and improve treatment outcomes for minority ethnic individuals, a multilevel approach combining interventions on an individual and systemic level is most likely required. In sum, our study presents a complex picture of help-seeking barriers among East-Africans with substance use problems in Norway. The findings indicate an urgent need for increased efforts to reduce the negative effects of stigma, racism, low health literacy and lack of culturally sensitive care.

### Strengths and limitations

A major strength of the current study is, considering the dearth of evidence on the subject, that it provides insight into the help-seeking experiences of migrant populations with substance use problems in Norway. Even though the sample was relatively small, the interviews provided rich data and sweeping narratives of the participants’ experiences. In IPA, small samples of up to six participants are preferred over larger samples due to each case’s demanding and iterative analysis [28]. The idiographic approach and modest requirements for sample size make IPA suitable for stigmatised or hard-to-reach populations. Furthermore, the study sample included two women previously described as difficult to recruit [21]. To our knowledge, ethnic minority women’s perspectives on help-seeking for substance use problems have not previously been explored in a Norwegian context, providing essential nuance to a complex subject matter. Recruitment assistance from patient organisation representatives and the first author’s clinical experience as a registered nurse in substance use care enabled trust during the interviews. The second author, a nurse, university professor and migrant from Africa, provided different perspectives than the first author, who is a white Norwegian graduate student. Both authors were involved in the analysis and interpretation of the data, reducing the influence of researcher bias.

A methodological challenge with conducting studies on migrant populations is the pitfall of treating citizens of different geographic, cultural and religious spheres as too homogenous and thereby risking ‘ethnic lumping’ [42]. We are mindful of the vast cultural, linguistic, historical and ethnic differences between and within the countries of Somalia, Eritrea and Sudan. However, it was necessary to balance the need for feasible recruitment from a hard-to-reach population and secure a homogenous sample relative to the experience under investigation. The interviewer has light skin and is working as a nurse in substance use care. It is possible that these factors might have influenced the participants’ willingness to share certain aspects of their experience.

### Conclusions and recommendations

This study shows that while the Norwegian healthcare system is grounded in the principle of health equality, healthcare is not equally accessible, nor does it provide the same quality of care to all citizens. Our findings indicate a need for more knowledge of mental health, substance use and equal access to health services for East African patients. Information about mental health, problem substance use, legal rights and health services navigation should be made available in more languages, and targeted information programmes should be directed towards migrant communities to combat stigma and increase mental health literacy. The inclusion of more specific health literacy outcomes in the Integration Act could trigger improved knowledge dispersion on mental health and substance use for newly arrived migrants.

Organisations and interest groups working to improve health equality for minority ethnic patients should be given more significant involvement in developing services and policies. Improved efforts should be made to recruit ethnic minority health personnel and individuals with treatment experience who can act as cultural brokers, decrease stigma and increase system trust in health care services. Increased use of interpreters could alleviate challenges of comprehension between health care personnel and patients with a migrant background. Evidence-based cultural competence development in healthcare professional education and healthcare services can be helpful to increase transcultural understanding and facilitate patient–professional encounters, but it is not sufficient to achieve the necessary systemic change. Moreover, considering that racism seems to be a contributing reason for health inequality in Norway, more research on the detrimental health effects of racism is needed in a Norwegian context to establish operational antiracist frameworks at the interpersonal and systemic levels.

## Data Availability

The data that support the findings of this study are available upon request from the corresponding author. The data are not publicly available due to information that could compromise the privacy of the research participants.
